# Identifying barriers to the administration of pre-hospital analgesia to adult trauma patients by UK paramedics: a qualitative interview study

**DOI:** 10.29045/14784726.2019.03.3.4.40

**Published:** 2019-03-01

**Authors:** Adam Smith, Jeremy Henning

**Affiliations:** Faculty of Health, Social Care and Education, a partnership between Kingston University and St George’s, University of London; Queen Mary’s University London

## Abstract

**Aims:**

The early delivery of effective analgesia is considered to be an important component of pre-hospital trauma care; however, the provision of analgesia by pre-hospital clinicians is often inadequate. While a number of studies have explored the underpinning attitudes and barriers to the paramedic administration of analgesia in various patient groups, specific barriers which prevent the paramedic administration of pre-hospital analgesia to adult trauma patients in the UK are poorly defined. The aim of this small study was to identify and define potential barriers to analgesia administration in this specific population. In doing so, this study will increase awareness and will form an important basis for future work aimed at improving the delivery of pre-hospital analgesia to adult trauma patients in the UK.

**Methods:**

Twenty paramedics employed in an urban ambulance service in the UK volunteered to participate and were recruited into this study. Semi-structured interviews were conducted with participants during 2018 in order to explore the potential barriers to analgesia administration. An inductive thematic analysis was undertaken on interview transcripts and potential barriers were defined from the themes identified, by reviewing the coded data within each theme.

**Results:**

All of the participants completed the study which identified 12 potential barriers to the paramedic administration of pre-hospital analgesia to adult trauma patients. These barriers were diverse in nature and related to factors including the patient presentation, paramedics’ perceptions of pain, the cautious use of analgesics, paramedics’ scope of practice, organisational procedures and clinical guidelines, as well as factors relevant specifically to the pre-hospital environment. Some of these barriers may be modifiable through improved paramedic education and training or by widening of the paramedic scope of practice, whereas others may not be modifiable.

There were a number of limitations to this study including the use of volunteer self-selection which may present a source of self-selection bias, the recruitment of paramedics from a single system which reduces the generalisability of the results and the absence of a second researcher.

**Conclusion:**

The identification of these potential barriers should form a basis for future work aimed at improving the paramedic delivery of pre-hospital analgesia to adult trauma patients. While many of the barriers identified may be present across pre-hospital care systems, some may be specific to the system in which this study was undertaken, to other urban systems or to paramedic practice in the UK.

